# The prevalence and mortality risks of PRISm and COPD in the United States from NHANES 2007–2012

**DOI:** 10.1186/s12931-024-02841-y

**Published:** 2024-05-15

**Authors:** Christopher J. Cadham, Hayoung Oh, MeiLan K. Han, David Mannino, Steven Cook, Rafael Meza, David T. Levy, Luz María Sánchez-Romero

**Affiliations:** 1 School of Public Health, Department of Health Management and Policy, University of Michigan, Ann Arbor, MI USA; 2 Georgetown University-Lombardi Comprehensive Cancer Center, Washington, DC USA; 3 Division of Pulmonary and Critical Care, University of Michigan Health System, Ann Arbor, MI USA; 4 Division of Pulmonary and Critical Care Medicine, University of Kentucky, Lexington, KY USA; 5 COPD Foundation, Miami, FL USA; 6 School of Public Health, Department of Epidemiology, University of Michigan, Ann Arbor, MI USA; 7 BC Cancer Research Institute, Vancouver, Canada

**Keywords:** Chronic obstructive pulmonary disease, Preserved ratio impaired spirometry

## Abstract

**Background:**

We estimated the prevalence and mortality risks of preserved ratio impaired spirometry (PRISm) and chronic obstructive pulmonary disease (COPD) in the US adult population.

**Methods:**

We linked three waves of pre-bronchodilator spirometry data from the US National Health and Nutritional Examination Survey (2007–2012) with the National Death Index. The analytic sample included adults ages 20 to 79 without missing data on age, sex, height, BMI, race/ethnicity, and smoking status. We defined COPD (GOLD 1, 2, and 3–4) and PRISm using FEV_1_/FVC cut points by the Global Initiative for Chronic Obstructive Lung Disease (GOLD). We compared the prevalence of GOLD stages and PRISm by covariates across the three waves. We estimated adjusted all-cause and cause-specific mortality risks by COPD stage and PRISm using all three waves combined.

**Results:**

Prevalence of COPD and PRISm from 2007–2012 ranged from 13.1%-14.3% and 9.6%-10.2%, respectively. We found significant differences in prevalence by sex, age, smoking status, and race/ethnicity. Males had higher rates of COPD regardless of stage, while females had higher rates of PRISm. COPD prevalence increased with age, but not PRISm, which was highest among middle-aged individuals. Compared to current and never smokers, former smokers showed lower rates of PRISm but higher rates of GOLD 1. COPD prevalence was highest among non-Hispanic White individuals, and PRISm was notably higher among non-Hispanic Black individuals (range 31.4%-37.4%). We found associations between PRISm and all-cause mortality (hazard ratio [HR]: 2.3 95% CI: 1.9—2.9) and various cause-specific deaths (HR ranges: 2.0–5.3). We also found associations between GOLD 2 (HR: 2.1, 95% CI: 1.7–2.6) or higher (HR: 4.2, 95% CI: 2.7–6.5) and all-cause mortality. Cause-specific mortality risk varied within COPD stages but typically increased with higher GOLD stage.

**Conclusions:**

The prevalence of COPD and PRISm remained stable from 2007–2012. Greater attention should be paid to the potential impacts of PRISm due to its higher prevalence in minority groups and its associations with mortality across various causes including cancer.

**Supplementary Information:**

The online version contains supplementary material available at 10.1186/s12931-024-02841-y.

## Introduction

Chronic obstructive pulmonary disease (COPD) is the third leading cause of death in the US [1–4]. The clinical diagnosis of COPD requires a spirometry FEV_1_/FVC ratio of less than 0.7, representing the ratio of the maximum amount of air that the subject can forcibly expel during the first second following maximal inhalation (FEV_1_) divided by the forced vital capacity (FVC). However, COPD is a progressive and chronic lung obstruction operating on a spectrum, and those not meeting the clinical COPD definition may still experience lung obstruction abnormalities.

Nomenclature for low lung function is heterogeneous [5]. Recently, Preserved Ratio Impaired Spirometry (PRISm), [6–9] defined as those with an FEV_1_/FVC ratio greater or equal to 0.7 and FEV_1_ less than 0.8, has been proposed as a pre-clinical COPD abnormal spirometry. PRISm replaces terms such as *Global Initiative for Chronic Obstructive Lung Diseases (GOLD)-Unclassified* and *restrictive spirometry* [9, 10]. PRISm serves as a more informative name that distinguishes the patterns of PRISm as different from restriction and non-specific abnormality [10]. PRISm is associated with increased cardiovascular mortality, physical strength limitations, higher body mass index (BMI), respiratory symptoms, diabetes, a history of stroke, and hypertension [4, 11–15]. However, those with PRISm are not easily categorized into a specific disease pathway given its comorbidities [7, 8, 14, 16] and the uncertainty of progression into a future COPD diagnosis [7, 8]. With only 22.2%-35.8% of individuals with PRISm expected to be diagnosed with COPD within five years, [17, 18] the causal pathway remains unclear, but those with PRISm are a population with poor health outcomes [6].

With an annual direct cost of $32 billion to the US healthcare system from COPD, [19] it is important to better understand the prevalence and mortality risks associated with different severity levels of abnormal spirometry. It is possible that addressing PRISm prior to a formal clinical COPD diagnosis could allow for preventative measures to help alleviate the physical burden of COPD for patients. Despite this, spirometry data are rarely collected in US population-based studies, making examinations of the prevalence and health risks of PRISm and COPD challenging.

The National Health and Nutrition Examination Survey (NHANES) Study collected spirometry data for three waves from 2007–2012. We used this data to examine the prevalence of PRISm and the different stages of COPD severity, and associations between PRISm and COPD severity and all-cause and cause-specific mortality using the NHANES Linked Mortality Files [20].

## Methods

### Study and data

Conducted by the National Center for Health Statistics at the US Centers for Disease Control and Prevention, NHANES is a nationally representative, cross-sectional survey of the civilian, non-institutionalized US population [21]. This study used three waves of data from NHANES: 2007–2008, 2009–2010, and 2011–2012 based on the availability of spirometry values. NHANES data were collected via household interviews and standardized physical examinations. Underrepresented subgroups such as Hispanic and Black populations, and low-income white persons were oversampled. The Asian racial/ethnic group was also oversampled in 2011–2012. NHANES sample selection and a more detailed survey description can be found elsewhere [22].

### Population

COPD and PRISm prevalence was estimated using a subset of US adults aged 20 to 79 eligible for spirometry tests [23]. Age limits of 20–79 were established based on Hankinson’s lung function predictive equation, which starts at ages 20 for males and 18 for women [24], and were capped at age 79 as the maximum eligible age for the NHANES spirometry. Response rate for the spirometry test was 77.3%, 87.3%, and 86.9% in 2007–2008, 2009–2010, and 2011–2012, respectively. The final prevalence analytic sample (*N* = 4,237 in 2007–2008, *N* = 4,783 in 2009–2010, and *N* = 4,308 in 2011–2012) included those with complete spirometry values and no-missing data for the lung function predictive equation or covariates (age, sex, height, BMI, race/ethnicity, and smoking status [*N* = 6,722 excluded (33%)]).

The final analytic sample for the mortality analyses (*N* = 13,307) included all participants who met the inclusion criteria for the prevalence analysis and had vital status ascertained through the NHANES Linked Mortality Files, which links NHANES records to the National Death Index [20].

### Spirometry, COPD, and PRISm definitions

The FVC, the FEV_1_, and FEV_1_/FVC were the spirometry values utilized for this analysis. Normal FEV_1_ values were estimated using Hankinson’s [24] gender-specific, non-Hispanic White predictive equations (FEV_1_ predicted for males = 0.5536–0.01303*age-0.000172*age^2 + 0.00014098*BMI^2 and FEV_1_ predicted for females = 0.4333–0.00361*age-0.000194*age^2 + 0.00011496*BMI^2) for all race/ethnicities following the methodology of similar studies [25].

We decided against Hankinson’s race-specific equations, which were created and validated for the NHANES III Study, [24] as there is growing evidence that use of race-specific equations the underestimates the prevalence of COPD across racial groups [26–29].

To maximize our analytic sample, COPD diagnosis was based on pre-bronchodilator spirometry values data (respondents with pre-bronchodilator *N* = 20,050, respondents with post-bronchodilator *N* = 1,564); an approach consistent with other studies [30]. We defined COPD as recommended by the Global Initiative for Chronic Obstructive Lung Disease (GOLD) as FEV_1_/FVC ratio of less than 0.7 [31] and further classified into GOLD stages based on FEV_1_ predicted values: GOLD 1 (FEV_1_ ≥ 80%), GOLD 2 (50% < FEV_1_ < 80%), and GOLD 3 + 4 (FEV_1_ ≤ 50%). Preserved ratio impaired spirometry (PRISm) was defined as those with an FEV_1_/FVC value of ≥ 0.7 but with abnormal spirometry (i.e., FEV_1_ < 80%) [16]. Individuals without PRISm or COPD were classified as GOLD 0 or normal spirometry.

### Covariates

Covariates were selected based on the inputs for the FEV_1_ predictive equations: age, gender, smoking status, body mass index (BMI) and race/ethnicity [7, 8, 14, 16]. For prevalence estimates, age was recoded as three age groups: 20–39, 40–64, and 65 + . Gender was defined as male or female. NHANES categorized race/ethnicity into five categories: non-Hispanic White, non-Hispanic Black, Mexican–American, Hispanic Other, and Other. For cigarette use status, we considered: “Have you smoked at least 100 + cigarettes in your lifetime?” and “Are you currently smoking?”. Current smokers smoked at least 100 + cigarettes in their lifetime and were currently smoking. Former smokers smoked at least 100 + cigarettes in their lifetime and did not currently smoke at the time of the survey. Never smokers were those who had not smoked 100 + lifetime cigarettes. BMI was categorized into three groups: normal (BMI < 25), overweight (25 ≤ BMI < 30), and obesity (BMI ≥ 30).

### Outcomes

For the prevalence analysis, COPD (Total, GOLD 1, GOLD 2 and GOLD 3–4) and PRISm were the outcomes of interest. For the mortality analysis, we estimated all-cause and cause-specific (i.e., cancer, cardiovascular disease, and chronic lower respiratory diseases) mortality for overall COPD, GOLD stages and PRISm.

### Statistical analysis

For each of the NHANES wave used, we calculated the weighted [32] population prevalence estimates for each of the outcomes, as well as the prevalence by age, gender, smoking status, and race/ethnicity using full sample mobile examination center exam weight. Differences in the prevalence of disease point estimates and 95% confidence intervals (95% CI) were compared across the three waves. Cox proportional hazard models were estimated to examine all-cause and cause-specific mortality risks for COPD (overall COPD, GOLD 1, GOLD 2 and GOLD 3–4) and PRISm, adjusting for age (both categorically and continuously in separate analyses), gender, BMI, smoking status, and race/ethnicity. Weights were adjusted for mortality analyses to enable the pooling of all three survey waves [33]. Analyses were performed using STATA 17.0 and R 4.3.2.

#### Sensitivity analyses

Given recent debates over the merits of race-specific predicative equations for spirometry, [28, 29, 34–36] in addition to Hankinson’s non-Hispanic White predictive equation of lung function, we also applied a race-neutral reference equation to explore differences in COPD prevalence and mortality. We used the Global Lung Function Initiative’s (GLI) race-neutral predictive equation for this secondary analysis [35]. The race-neutral equation removes race-specific adjustments which may bias spirometry results and reflects the wide variation of lung function within and between populations.

In this secondary analysis, we estimated COPD (Total, GOLD 1, GOLD 2 and GOLD 3–4) and PRISm prevalence and all-cause mortality for COPD GOLD stages and PRISm. We also plotted the estimates from the GLI race-neutral predictive equation compared to the respective outcomes using the Hankinson non-Hispanic White predictive equation.

## Results

Table 1 presents the weighted demographics and COPD prevalence for 2007–2012 and each wave individually. Characteristics of the sample remained stable over the period.

**Table 1:** Sample characteristics for each NHANES wave

	**Overall 2007–2012 (*N* = 13,328)**		**2007–2008 (*N* = 4237)**		**2009–2010 (*N* = 4783)**		**2011–2012 (*N* = 4308)**	
	**%(N)**	95% CI	**%(N)**	95% CI	**%(N)**	95% CI	**%(N)**	95% CI
**Age (years)**								
20–39	39.7% (5009)	37.5%—42.0%	40.5 (1529)	37.3%—43.7%	39.5 (1787)	36.7%—42.2%	39.2 (1693)	34.0%—44.7%
40–64	48.1% (6071)	46.2%—49.9%	48 (1935)	45.5%—50.6%	47.8 (2180)	45.5%—50.1%	48.4 (156)	43.9%—53%
65 +	12.2% (2248)	11.4%—13.1%	11.5 (773)	10.0%—13.1%	12.7 (816)	11.4%—14.2%	12.4 (659)	11.1%—13.9%
**Gender**								
Male	49.5% (6650)	48.6%—50.4%	49.6% (2131)	48.2%—51.1%	49.6% (2353)	48.3%—51%	49.1% (2166)	47.2%—51.2%
Female	50.5% (6678)	49.6%—51.4%	50.4% (2106)	49%—51.8%	50.4% (2430)	49%—51.7%	50.9% (2142)	48.8%—52.8%
**Smoking Status**								
Current	21.7% (3030)	20.3%—23.1%	23.8% (1016)	20.8%—27.1%	20.9% (1102)	19.3%—22.6%	20.5% (912)	18%—23.2%
Former	23.3% (2999)	21.8%—24.9%	23.7% (1017)	22.1%—25.4%	23% (1069)	20.1%—26.4%	23.2% (913)	20.1%—26.6%
Never	55.0% (7299)	53.0%—57.1%	52.5% (2204)	48.5%—56.4%	56.1% (2612)	52.2%—59.9%	56.4% (2483)	53.1%—59.6%
**Race/Ethnicity**								
Mexican	8.4% (2108)	6.5%—10.7%	8.5% (768)	6%—12%	8.8% (905)	5.1%—14.6%	7.8% (434)	4.8%—12.4%
Other Hispanic	5.6% (1436)	4.2%—7.3%	4.9% (481)	3%—7.9%	5.3% (521)	3.1%—8.9%	6.5% (434)	4%—10.3%
NH White	68.0% (5698)	63.5%—72.1%	70.1% (1970)	61.9%—77.3%	67.8% (2231)	60.4%—74.5%	66.2% (1497)	57.2%—74.1%
NH Black	11.3% (2911)	9.3%—13.5%	10.7% (852)	7.2%—15.5%	11.3% (876)	9.5%—13.3%	11.8% (1183)	7.8%—17.4%
Other	6.9% (1175)	5.7%—8.3%	5.9% (116)	3.8%—8.9%	6.8% (249)	5%—9.3%	7.8% (760)	5.7%—10.7%
**COPD Severity**								
GOLD 0	76.4% (9677)	75.2%—77.6%	76.2% (3072)	74.1%—78.1%	77.25% (3565)	75.2%—79.2%	75.8% (3040	73.3%—78.1%
PRISm	9.8% (1862)	8.9%—10.8%	9.6% (536)	8.1%—11.4%	9.6% (593)	7.9%—11.7%	10.2% (733)	8.7%—11.9%
GOLD 1	6.9% (810)	6.2%—7.7%	7.1% (287)	6.2%—8.3%	7.2% (299)	5.7%—9.1%	6.5% (224)	5.4%—7.8%
GOLD 2	6.0% (836)	5.4%—6.6%	6.3% (300)	5.2%—7.5%	5.3% (277)	4.6%—6%	6.5% (259)	5.3%—7.9%
GOLD 3–4	0.9% (143)	0.7%—1.1%	0.9% (42)	0.5%—1.4%	0.7% (49)	0.4%—1.1%	1.2% (52)	0.8%—1.6%
COPD (GOLD 1–4)	13.8% (1789)	12.8%—14.9%	14.3% (629)	12.4%—16.3%	13.1% (624)	11.2%—15.4%	14.1% (535)	12.3%—16%

### Prevalence of COPD and PRISm

**Fig. 1:**
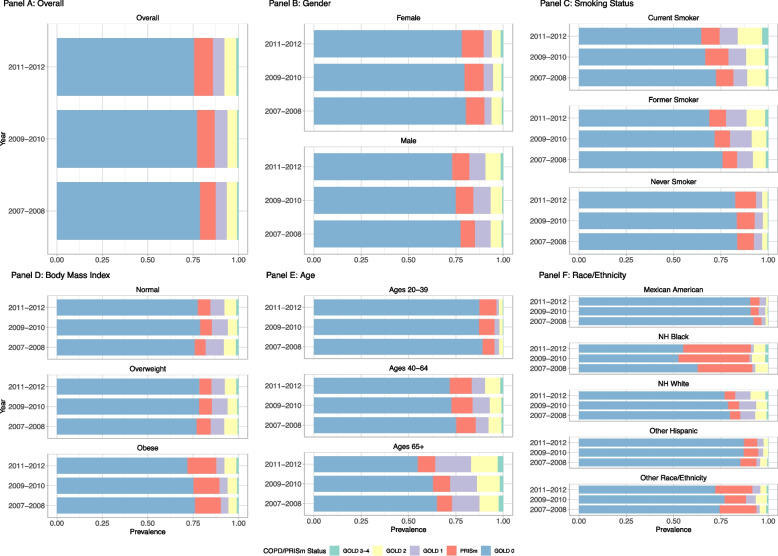
COPD and PRISm prevalence by covariates and cohort waves. Legend: Figure 1 presents prevalence of each COPD GOLD stage and PRISm by subgroup and wave of NHANES. Point estimates and confidence intervals are available in Table 1 (overall estimates) and Appendices 2–6 (subgroups)

The overall prevalence estimates for COPD and PRISm are available in Table 1 and Fig. 1: Panel A. Across all three waves, the prevalence of COPD was 13.8% (95% CI: 12.8%—14.9%) and PRISm 9.8% (95% CI: 8.9%—10.8%). Trends in COPD remained relatively stable from 2007 to 2012. Similar stability was seen among participants with PRISm. Prevalence estimates of COPD using spirometry were substantially higher than self-reported COPD at 5.9% (95% CI: 5.0%—6.9%) for 2007–2012 (Appendix 1).

### Prevalence of COPD and PRISm by gender, smoking status, BMI, age group, and race/ethnicity

Estimates of COPD and PRISm by gender show (Fig. 1: Panel B; Appendix 2) that overall COPD was statistically significantly higher in males (range: 17.4%—17.9%), while PRISm was higher among females (range 10.5%—11.3%), albeit not significantly, for all survey waves. However, we did not observe significant differences in disease stages (Appendix 2).

Overall, the prevalence of COPD was statistically significantly higher among current and former cigarette users compared to never users (Fig. 1: Panel C; Appendix 3). PRISm estimates were highest among current users for all years except 2011–2012 when the highest prevalence was among never users (10.9%, 95% CI: 8.7%—11.3%), but differences between groups were not statistically significant. Former users had the lowest rates of PRISm across all three waves of data, but the highest rates of GOLD 1 (range: 10.2%-11.6%), while current users reported the highest rates of GOLD 2 (range: 11.8%—12.9%) and GOLD 3–4 (range: 1.5%—3.1%).

COPD prevalence was lower for individuals with obesity compared to those with a BMI categorized as normal or overweight (Fig. 1: Panel D, Appendix 4). The differences in prevalence were particularly notable for GOLD 1, which was roughly twice as high among normal-weight individuals compared to those with obesity. PRISm estimates across all three waves were higher for people with obesity than people with a normal BMI.

Estimates by age group showed increases in COPD with age and reported the highest prevalence among ages 65–79 (range: 28.1%-36.1%) (Fig. 1: Panel E; Appendix 5). Conversely, PRISm prevalence was highest among ages 40–64 (range: 11.0%—11.9%) and lowest at ages 20–39 (range: 6.6%—8.9%).

Distinct patterns were observed for prevalence estimates by race/ethnicity (Fig. 1: Panel F; Appendix 6). PRISm prevalence was higher among Non-Hispanic Black participants (range: 31.4%—37.4%), followed by those classified as having other race/ethnicity (range: 11.2%—20.4%). Conversely, non-Hispanic White participants had the highest prevalence of COPD across all three survey waves (range: 15.5%—17.5%), with GOLD 1 making up roughly half of those cases (range: 8.2%—9.3%). The prevalence of COPD and PRISm were roughly equal among Mexican Americans.

### Mortality estimates

**Fig. 2:**
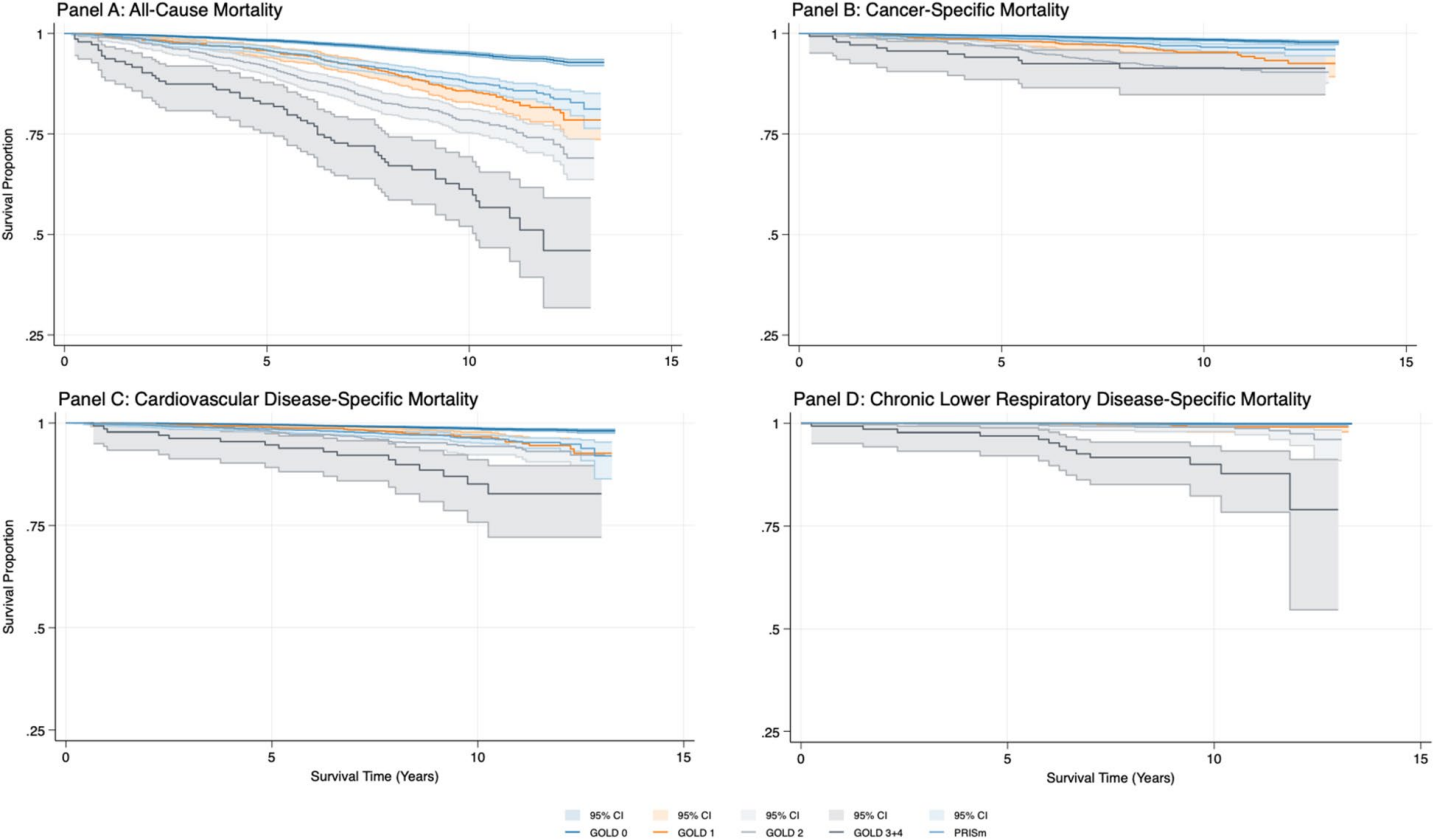
COPD and PRISm survival curves by mortality outcome. Legend: Figure 2 presents Kaplan–Meier curves for each survival outcome by COPD GOLD stage and PRISm. Curves show the proportion of individuals still alive at a given time point. Shaded areas represent 95% confidence intervals

Of the 13,307 participants in the mortality analysis, 1,079 (8.15%) died, with a median follow-up of 9.4 years. In multivariable models adjusting for gender, race/ethnicity, age at screening exam, BMI, and smoking status, the risk of all-cause mortality was higher among those at GOLD 2 (HR: 2.1 95% CI: 1.7–2.6) or higher (HR: 4.2, 95% CI: 2.7–6.5) and for those at PRISm (HR: 2.3, 95% CI: 1.9–2.9) compared to GOLD 0 (Fig. 2, Table 2).

**Table 2:** Cox proportional hazard models for all-cause and cause-specific mortality for NHANES 2007–2012

	All-Cause Mortality		Cancer		Cardiovascular Diseases		Chronic Lower Respiratory Diseases	
	Hazard Ratio	95% CI	Hazard Ratio	95% CI	Hazard Ratio	95% CI	Hazard Ratio	95% CI
**COPD Status**								
GOLD 0	Ref		Ref		Ref		Ref	
PRISm	**2.3**	**(1.9—2.9)**	**2.0**	**(1.2—3.2)**	**2.4**	**(1.6—3.6)**	**5.3**	**(1.1—26.0)**
GOLD 1	1.2	(0.9—1.6)	1.4	(0.9—2.4)	0.9	(0.6—1.4)	1.4	(0.4—5.4)
GOLD 2	**2.1**	**(1.7—2.6)**	**2.0**	**(1.4—2.8)**	**2.4**	**(1.6—3.7)**	3.3	(1.0—11.3)
GOLD 3–4	**4.2**	**(2.7—6.5)**	1.3	(0.6—2.8)	**4.7**	**(2.3—9.8)**	**53.2**	**(14.0—202.0)**
**Gender**								
Female	Ref		Ref		Ref		Ref	
Male	**1.7**	**(1.5 – 2.0)**	**1.7**	**(1.3—2.4)**	**2.1**	**(1.5—3.1)**	1.6	(0.7—3.6)
**Age**								
Age 20–39	**0.3**	**(0.2—0.4)**	**0.1**	**(0.0—0.3)**	**0.2**	**(0.1—0.4)**	**0.1**	**(0.0—0.9)**
Age 40–64	Ref		Ref		Ref		Ref	
Age 65 +	**4.7**	**(4.0—5.5)**	**4.3**	**(3.1—6.0)**	**6.6**	**(4.9—8.9)**	**4.3**	**(1.3—13.7)**
**Body Mass Index**								
Normal	Ref		Ref		Ref		Ref	
Overweight	0.9	(0.7—1.1)	0.8	(0.5—1.3)	1.3	(0.8—2.1)	0.5	(0.2—1.8)
Obese	1.1	(0.9—1.4)	1.0	(0.7—1.4)	**1.8**	**(1.1—2.7)**	0.9	(0.2—3.3)
**Smoking Status**								
Never Smoker	Ref		Ref		Ref		Ref	
Current Smoker	**2.1**	**(1.7—2.6)**	**2.3**	**(1.4—3.6)**	**1.6**	**(1.1—2.3)**	**5.8**	**(1.6—21.7)**
Former Smoker	1.2	(0.9—1.4)	1.1	(0.7—1.7)	0.9	(0.7—1.3)	3.0	(0.9—9.9)
**Race/Ethnicity**								
Non-Hispanic White	Ref		Ref		Ref		Ref	
NH Black	1.0	(0.8—1.2)	0.9	(0.6—1.3)	1.0	(0.7—1.5)	0.4	(0.2—0.9)
Mexican American	0.8	(0.6—1.0)	0.6	(0.4—1.1)	0.9	(0.6—1.5)	0.4	(0.0—3.5)
Other Hispanic	0.8	(0.6—1.1)	0.8	(0.5—1.2)	1.1	(0.6—1.8)	0.1	(0.0—1.1)
Other	**0.6**	**(0.4—0.8)**	0.6	(0.3—1.3)	0.6	(0.3—1.0)	1.0	(0.2—4.4)

Values in **bold** are statistically significant

COPD stages and PRISm were also significant predictors of cause-specific mortality from cancer, cardiovascular disease, and chronic lower respiratory diseases. GOLD 2 was associated with a significant risk of cancer mortality (HR: 2.0, 95% CI: 1.4–2.8), as was PRISm (HR: 2.0, 95% CI: 1.2–3.2) compared with GOLD 0. GOLD3 + 4 (HR: 4.7, 95% CI: 2.3–9.8) showed the highest risk of cardiovascular mortality, followed by GOLD 2 (HR: 2.4, 95% CI: 1.6–3.7) and PRISm (HR: 2.4, 95% CI: 1.6–3.6) which conferred similar cardiovascular mortality risks. Finally, GOLD 3 + 4 (HR: 53.2, 95% CI: 14.0–202.0) and PRISm (HR: 5.3, 95% CI: 1.1–26.0) were both associated with an increased risk of chronic lower respiratory disease-related mortality when compared to GOLD 0.

We found no differences in estimates when using continuous age compared to categorical with the exception of the risk of mortality from chronic lower respiratory diseases among individuals with PRISm which was no longer statistically significant (Appendix 7).When comparing PRISm to any COPD (Appendix 8 and 9), mortality risks from PRISm trended higher than those of any COPD except for chronic lower respiratory disease-specific mortality.

### Race-neutral predictive equation

**Fig. 3:**
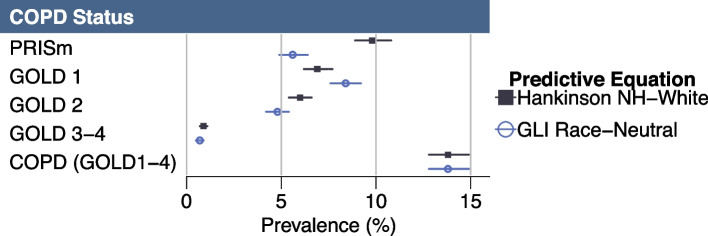
Comparison of COPD GOLD stage and PRISm prevalence estimates using Hankinson’s non-Hispanic White and the GLI race-neutral predictive equations. Legend: Figure 3 presents a forest plot that displays prevalence estimates and 95% confidence intervals of COPD GOLD stages and PRISm using weighted NHANES data from 2007–2012. These results are presented in tabular form in Appendix 10

When using the Global Lung Function Initiative (GLI) Race-Neutral predictive equation (Fig. 3, Appendix 10), we find significantly lower estimates of PRISm. Both equations led to the same overall COPD prevalence. While we found no statistically significant differences in COPD GOLD stage between the two equations, there may be downstaging with the race-neutral equation. The race-neutral equation results show higher point-estimates for GOLD 1, and lower point-estimates for GOLD 2 and 3–4 relative to the Hankinson equation.

Use of the race-neutral equation resulted in no statistically significant differences mortality outcomes (Appendices 11 and 12).

## Discussion

In a nationally representative dataset of US adults, our study found that from 2007–2012, COPD and PRISm trends were stable. The average prevalence of PRISm was 9.8% and 13.8% for COPD. Using race-neutral predictive equations, we found lower estimates of PRISm, but consistent estimates of COPD. By GOLD stages, we found that the prevalence of GOLD 1 was 6.9%, 6.0% for GOLD 2, and 0.9% for GOLD 3–4. Both COPD and PRISm were associated with greater all-cause mortality, with greater mortality risk at higher COPD stages. Further, despite having different demographic and cigarette smoking profiles, both COPD stages and PRISm were associated with an increased risk of cause-specific mortality outcomes, including cancer- and cardiovascular disease-specific mortality.

Our PRISm prevalence estimates were generally consistent with those reported in other national and international studies. Using data from the US National Heart, Lung, and Blood Institute (NHLBI) pooled cohort study, Wan et al. reported a PRISm prevalence of 8.5% between 1975–2018. Similarly, researchers found a PRISm prevalence of 11.0% in a United Kingdom population between 2006–2013, [37] while a cohort study from Rotterdam, Netherlands found a PRISm prevalence of 7.6% [4]. These prevalence estimates highlight the importance of monitoring PRISm trends using nationally representative surveys. Left unattended, PRISm could contribute to the increasing global chronic obstructive respiratory disease burden.

In the US, there is a lack of COPD nationally representative prevalence estimated from spirometric values. This is partly due to NHANES being the only national health survey that collected spirometric values among those who collect COPD-associated data (i.e., the Population Assessment of Tobacco and Health, the Behavioral Risk Factor Surveillance System, and the National Health Interview Survey). Still, comparisons of our spirometric COPD estimates and those obtained from self-reported COPD data show that COPD prevalence as defined by spirometry is almost double that from self-reported measures. From 2011 to 2020, the Behavioral Risk Factor Surveillance System reported a stable prevalence of around 6.0% [38]. Another study using NHANES self-reported data from 2007–2012 reported a COPD prevalence of 5.2% [39]. Similarly, we performed a sensitivity analysis using self-reported defined COPD from NHANES 2007–2012 and estimated a prevalence of 5.9% across this period (Appendix 6). Reliance on self-reported measures appears to underestimate the burden of COPD systematically. It has previously been reported that a substantial number of individuals, nearly 80%, with spirometry-defined COPD cannot be identified with self-reported questionnaires [40, 41]. Furthermore, studies that compare self-report and spirometry results suggest that those with more regular contact with the healthcare system are more likely to be diagnosed with COPD, [39] which may bias prevalence estimates to those with better access to medical care.

Our stratified findings by sub-group covariates also revealed similar results to those in the literature. Self-report studies have suggested limited differences in COPD by gender, yet females may be more likely to be diagnosed with COPD [39, 42]. However, using spirometry to define COPD, we found that females have a lower prevalence of COPD and a higher prevalence of PRISm than males. These results are consistent with previous studies that find a faster decline in FEV_1_ among female smokers and overall increases in COPD prevalence among women [43, 44]. The observed higher PRISm prevalence estimates among females than males could potentially reflect the faster decline in lung function.

As with previous studies, our estimates show that COPD prevalence increased by age group [45–47]. However, this was not the case for PRISm, with the highest prevalence at ages 40–64. Other studies in the US have also reported that a higher prevalence of PRISm is found in subjects aged 45 to 68 [16, 48]. Those with PRISm in middle ages might transition to COPD before reaching age 65, therefore “diluting” the prevalence of PRISm at older ages as FEV_1_ and FVC decline at different rates. Alternatively, individuals with PRISm may die at younger ages due to the increased mortality risk of PRISm [49]. Still, more evidence on the natural history of COPD and the role of PRISm in developing COPD is necessary.

Our results found that current cigarette users had the highest COPD prevalence, followed by former smokers and then never smokers, which is also consistent with previous literature [42, 50]. While smoking is an established risk factor for COPD, other risk factors such as asthma and hazardous work conditions contribute to the prevalence of COPD among never smokers [51]. As with other sub-group categories, PRISm prevalence did not follow the same patterns as COPD when stratified by smoking status. Current and never users had higher PRISm prevalence, with former smokers having the lowest. This pattern might also be explained by the transition of PRISm to COPD.

COPD has been strongly associated with BMI. Our findings add to the evidence which shows, regardless of the COPD definition (i.e., self-reported or spirometry), that individuals with obesity showed a lower COPD prevalence. Published studies have reported that overweight and obese individuals are less likely to have GOLD stage 3–4 than individuals with a normal BMI [52]. This may be due in part to lower FVC and FEV_1_ among people with obesity [53]. Alternatively, COPD patients with obesity report more symptoms and poorer quality of life than patients with a normal weight, [54] which may lead undiagnosed individuals with obesity to seek treatment earlier than those with normal BMI. Coupled with receiving more intense treatment, [55] those with obesity may see improved lung function. Previous studies have also found a substantially higher prevalence of PRISm among people with higher BMI [16].

COPD and PRISm prevalence stratified by race yielded several interesting results. Generally, the highest COPD prevalence was among non-Hispanic Whites, followed by non-Hispanic Blacks, those of other race/ethnicity, non-Mexican–American Hispanic individuals, and those in the Mexican–American racial category. Of note, non-Hispanic Blacks had the highest PRISm prevalence (30% +), resulting in a difference of around 20 percentage points between PRISm and COPD prevalence for this group. Previous studies have found inconsistent results on the relationship between race and PRISm/COPD prevalence [48, 57]. More work is necessary to identify the extent to which racial/ethnic differences in COPD and PRISm prevalence exist and the root causes of these differences.

Through our survival analysis, we found that COPD and PRISm were both significantly associated with an increased mortality risk. PRISm was a consistent indicator of increased mortality risk from cancer, cardiovascular disease, and chronic lower respiratory disease, despite being comprised of fewer current and former smokers than COPD states.

When combined with findings from various countries and data sources, the results from our nationally representative study provide compelling evidence of the impact of COPD and PRISm on mortality. The increased mortality risk from PRISm has been corroborated by Yang et al., whose meta-analysis reports an increased risk of all-cause cardiovascular and respiratory-related mortality compared to normal spirometry [49]. In the US, Wan et al., reported that PRISm diagnosis is associated with an increased risk for all-cause, cardiovascular and respiratory mortality compared to individuals with normal spirometry using the National Heart, Lung and Blood Institute Pooled Cohort Study, a non-nationally representative sample [48]. Similar risks have also been reported for all-cause mortality in the UK using data from the UK BioBank, [37] for all-cause and cardiovascular mortality in the Netherlands, [4] and for all-cause and cardiovascular mortality in Japan [58].

Less is known of the impacts of PRISm on cancer mortality. Our study’s results indicating a significant impact are the first such finding in a US general population. Nevertheless, these findings align with research from South Korea which found PRISm increased the risk of mortality from lung cancer [59].

Emerging evidence suggests that, like COPD, PRISm is a heterogeneous condition. Studies have indicated that PRISm can be categorized into restrictive PRISm (FEV1/FVC ≥ 0.7, FEV1 < 80%, and FVC < 80%) and non-restrictive PRISm (FEV1/FVC ≥ 0.7, FEV1 < 80%, and FVC ≥ 80%). This variability extends to differences in risk factors, [60] the likelihood of transitioning to airflow obstruction, exacerbations, and mortality risks for all-cause and cardiovascular mortality [61–63]. Therefore, future efforts should prioritize improving diagnostic approaches and mortality risks for individuals with this condition.

For COPD, our results show an increased risk of all-cause mortality as well as cardiovascular disease-specific mortality for GOLD 2 or higher. While we found evidence of significant increases in the risk of chronic lower respiratory disease-related mortality among GOLD 3–4 patients, the smaller number of individuals in this group limits confidence in these estimates, as is reflected in the large confidence intervals. Notably, we consistently find higher risk of mortality with PRISm than with GOLD 1 which questions the hypothesis that PRISm serves as a precursor state to COPD.

In recent years, a substantive body of research has demonstrated the bias associated with use of race-specific predictive equations [26, 29, 34, 36]. As such, we avoided the use of race-specific equations in all our analyses and opted to use Hankinson’s non-Hispanic White predictive equation for all races and the GLI race-neutral predictive equation in a secondary analysis. Apart from lower rates of PRISm using the GLI equation, we find no statistically significant differences in COPD prevalence between these two approaches. However, the lower rates of PRISm with race-neutral equations, as well as higher trends of lower stages of COPD may suggest that race-neutral equations are downstaging patients. Regardless, this did not have impacts on mortality estimates. More research is needed, particularly evaluating the precision of race-neutral equations for the identification of PRISm.

This study has several limitations. First, our prevalence data are cross-sectional, and we cannot make inferences regarding the causal pathways of PRISm and COPD. Second, spirometry data were only available in the NHANES data between 2007–2012, limiting our ability to examine longer-term and more recent trends in COPD and PRISm prevalence as spirometry examination by NHANES was discontinued in 2013. Enhancing future research requires the collection of longitudinal spirometric data alongside additional nationally representative cross-sectional data. Broadening datasets to encompass spirometry results that reflect the current trends and diversity of the disease across the nation is essential. Third, we used pre-bronchodilator spirometry values due to sample size limitations for post-bronchodilator values in this study. While some research has found a non-significant discrepancy between the use of pre-bronchodilator or post-bronchodilator for air obstruction diagnosis, GOLD standards recommend the use of post-bronchodilator data [64, 65]. Although there is still controversy on the estimated outcomes from each measure [66]. Previous studies using the NHANES data have been published with the same limitation, [11, 15] and nationally representative data with post-bronchodilator data are needed. Furthermore, we defined COPD based on an FEV_1_/FVC ratio of 0.7, as is commonly done in research, rather than the lower limit of normal (LLN). FEV_1_/FVC ratio is age-dependent, and its utilization may result in misdiagnosed airflow obstruction [68]. Consequently, there is a possibility that our analysis may overestimate the prevalence of PRISm among younger individuals and underestimate PRISm among older individuals. Finally, we did not account for the potential impacts of measurement error in the spirometry [68].

## Conclusion

Using a nationally representative sample of US adults with spirometry data between 2007–2012, we found that approximately 10% of the US adult population meet the criteria for PRISm, and 14% meet the criteria for COPD. Moreover, both PRISm and COPD were associated with an increased risk of all-cause and cause-specific mortality, demonstrating the importance of examining the potential impact of PRISm at the national level. Much remains unknown about PRISm and the extent to which it may progress to a formal COPD diagnosis; as such, increasing spirometric data collection is essential.

### Supplementary Information

Supplementary Material 1.

## Data Availability

All data used in this study is publicly available from the US Centers for Disease Control and Prevention through the National Center for Health Statistics which manages NHANES.
