# Supplementary material for: The prevalence and mortality risks of PRISm and COPD in the United States from NHANES 2007–2012
Source: Respir Res. 2024 May 15;25:208. doi: 10.1186/s12931-024-02841-y (PMC11096119; doi:10.1186/s12931-024-02841-y)
Supplement: Supplementary file 1 — Supplementary Material 1. [file 12931_2024_2841_MOESM1_ESM.docx]

**Appendix Table 1: Self-reported COPD from NHANES 2007-2012**

Self-reported COPD was defined as the participant said “Yes” to the question "Has a doctor or other health professional ever told you that you had chronic bronchitis OR emphysema?”

|  | **Prevalence (%)** | **95% CI** |
| --- | --- | --- |
| **Overall (2007-2012)** | 5.9 | 5.0-6.9 |
| 2007-2008 | 6.3 | 4.7-8.7 |
| 2009-2010 | 4.6 | 3.6-6.0 |
| 2011-2012 | 6.7 | 5.2-8.6 |

**Appendix Table 2: NHANES COPD and PRISm prevalence estimates stratified by gender from 2007-2012**

| **NHANES 2007-2008** | **Male** | **95% CI** | **Female** | **95% CI** |
| --- | --- | --- | --- | --- |
| **COPD & PRISm** |  |  |  |  |
| Prism | 8.7% (N=245) | 7.0% - 10.7% | 10.5% (N=291) | 8.7% - 12.7% |
| GOLD 1 | 9.9% (N=203) | 8.6% - 11.4% | 4.4% (N=84) | 3.4% - 5.8% |
| GOLD 2 | 6.6% (N=172) | 4.9% - 8.8% | 5.9% (N=128) | 4.4% - 8.0% |
| GOLD 3-4 | 1% (N=24) | 0.4% - 2.2% | 0.8% (N=18) | 0.5% - 1.2% |
| COPD (GOLD 1-4) | 17.4% (N=339) | 14.7% - 20.5% | 11.1% (N=230) | 9.2% - 13.5% |
| **NHANES 2009-2010** | **Male** | **95% CI** | **Female** | **95% CI** |
| **COPD & PRISm** |  |  |  |  |
| Prism | 9.2% (N=282) | 7.0% - 12.2% | 10% (N=311) | 8.5% - 11.7% |
| GOLD 1 | 9.3% (N=207) | 7.6% - 11.4% | 5.1% (N=92) | 3.4% - 7.7% |
| GOLD 2 | 6% (N=165) | 4.9% - 7.3% | 4.5% (N=112) | 3.5% - 5.9% |
| GOLD 3-4 | 0.5% (N=25) | 0.3% - 1.1% | 0.8% (N=24) | 0.5% - 1.5% |
| COPD (GOLD 1-4) | 15.8% (N=397) | 13.6% - 18.3% | 10.5% (N=228) | 8.2% - 13.3% |
| **NHANES 2011-2012** | **Male** | **95% CI** | **Female** | **95% CI** |
| **COPD & PRISm** |  |  |  |  |
| Prism | 9% (N=327) | 7.4% - 11.0% | 11.3% (N=406) | 9.3% - 13.6% |
| GOLD 1 | 8.6% (N=154) | 6.1% - 12.0% | 4.4% (N=70) | 3.0% - 6.5% |
| GOLD 2 | 8.1% (N=164) | 6.0% - 11.0% | 4.8% (N=95) | 3.6% - 6.4% |
| GOLD 3-4 | 1.2% (N=27) | 0.6% - 2.2% | 1.2% (N=25) | 0.5% - 2.7% |
| COPD (GOLD 1-4) | 17.9% (N=345) | 14.4% - 21.9% | 10.4% (N=190 | 8.0% - 13.4% |

**Appendix Table 3: NHANES COPD and PRISm prevalence estimates stratified by smoking status from 2007-2012**

| **NHANES 2007-2008** | **Current** | **95% CI** | **Former** | **95% CI** | **Never** | **95% CI** |
| --- | --- | --- | --- | --- | --- | --- |
| **COPD & PRISm** | |  |  |  |  |  |
| Prism | 11% (N=144) | 7.8% - 15.4% | 8.8% (N=125) | 7% - 11.1% | 9.3% (N=267) | 7.6% - 11.3% |
| GOLD 1 | 8.9% (N=83) | 6.8% - 11.6% | 10.2% (N=111) | 8.1% - 12.8% | 4.9% (N=93) | 3.6% - 6.7% |
| GOLD 2 | 11.8% (N=128) | 9.4% - 14.5% | 8% (N=99) | 5.8% - 10.6% | 3% (N=73) | 2.1% - 4.2% |
| GOLD 3-4 | 1.5% (N=15) | 0.6% - 3.5% | 1.4% (N=17) | 0.7% - 2.6% | 0.4% (N=10) | 0.2% - 0.9% |
| COPD (GOLD 1-4) | 22.1% (N=226) | 18.8% - 25.8% | 19.7% (N=227) | 16.2% - 23.6% | 9.3% (N=176) | 6.3% - 10.8% |
| **NHANES 2009-2010** | **Current** | **95% CI** | **Former** | **95% CI** | **Never** | **95% CI** |
| **COPD & PRISm** | |  |  |  |  |  |
| Prism | 12.1% (N=162) | 9.3% - 15.7% | 8.1% (N=121) | 6.4% - 10.1% | 9.3% (N=310) | 7.4% - 11.3% |
| GOLD 1 | 9.5% (N=89) | 7.2% - 12.5% | 11.6% (N=110) | 9.3% - 14.4% | 4.6% (N=100) | 3.4% - 6.1% |
| GOLD 2 | 9.9% (N=116) | 8.2% - 11.9% | 7.7% (N=86) | 6% - 9.7% | 2.5% (N=75) | 2% - 3.2% |
| GOLD 3-4 | 1.7% (N=26) | 1.1% - 2.5% | 0.9% (N=15) | 0.3% - 2.7% | 0.2% (N=8) | 0.1% - 0.6% |
| COPD (GOLD 1-4) | 21.1% (N=121) | 17.9% - 24.6% | 20.2% (N=211) | 16.8% - 24% | 7.3% (N=183) | 5.7% - 9.3% |
| **NHANES 2011-2012** | **Current** | **95% CI** | **Former** | **95% CI** | **Never** | **95% CI** |
| **COPD & PRISm** | |  |  |  |  |  |
| Prism | 9.6% (N=149) | 8.2% - 11.3% | 8.7% (N=122) | 6.3% - 11.9% | 10.9% (N=462) | 8.7% - 11.3% |
| GOLD 1 | 9.7% (N=70) | 8% - 11.8% | 11% (N=82) | 7.9% - 15% | 3.4% (N=72) | 2.5% - 4.7% |
| GOLD 2 | 12.9% (N=95) | 10.2% - 16.1% | 9.8% (N=82) | 6.6% - 14.2% | 2.8% (N=81) | 2% - 3.7% |
| GOLD 3-4 | 3.1% (N=22) | 1.8% - 5.3% | 1.6% (N=17) | 0.7% - 3.6% | 0.3% (N=13) | 0.1% - 0.7% |
| COPD (GOLD 1-4) | 25.7% (N=187) | 21.3% - 30.7% | 22.3% (N=181) | 18.4% - 26.8% | 6.4% (N=166) | 5.2% - 7.9% |

**Appendix Table 4: NHANES COPD and PRISm prevalence estimates stratified by BMI from 2007-2012**

| **NHANES 2007-2008** | **Normal** | **95% CI** | **Overweight** | **95% CI** | **Obesity** | **95% CI** |
| --- | --- | --- | --- | --- | --- | --- |
| **COPD & PRISm** | |  |  |  |  |  |
| Prism | 6.2%, (N=94) | 4.9% - 7.8% | 7.9%, (N=150) | 6.7% - 9.4% | 14.5%, (N=292) | 11.5% - 18.2% |
| GOLD 1 | 9.9%, (N=115) | 7.4% - 13.3% | 7.5%, (N=108) | 6% - 9.4% | 4.1%, (N=64) | 2.7% - 6.1% |
| GOLD 2 | 6.7%, (N=107) | 4.6% - 9.6% | 7.4%, (N=103) | 5.8% - 9.3% | 4.7%, (N=90) | 3.7% - 6% |
| GOLD 3-4 | 1.4%, (N=14) | 0.7% - 2.8% | 0.4%, (N=10) | 0.2% - 0.9% | 0.8%, (N=18) | 0.5% - 1.5% |
| COPD (GOLD 1-4) | 18.0%, (N=236) | 14.7% - 22.0% | 15.3%, (N=221) | 12.4% - 18.7% | 9.7%, (N=172) | 7.9% - 11.7% |
| **NHANES 2009-2010** | **Normal** | **95% CI** | **Overweight** | **95% CI** | **Obesity** | **95% CI** |
| **COPD & PRISm** | |  |  |  |  |  |
| Prism | 6.7%, (N=112) | 5.4% - 8.3% | 7.1%, (N=152) | 4.7% - 10.6% | 14.4%, (N=329) | 11.4% - 18.1% |
| GOLD 1 | 8.8%, (N=116) | 6% - 12.6% | 8.8%, (N=116) | 6.7% - 11.3% | 4.5%, (N=67) | 3.4% - 5.9% |
| GOLD 2 | 5.2%, (N=81) | 4.1% - 6.6% | 5.3%, (N=94) | 4.1% - 6.9% | 5.3%, (N=102) | 4.1% - 6.7% |
| GOLD 3-4 | 0.7%, (N=14) | 0.2% - 1.8% | 0.6%, (N=14) | 0.3% - 1.2% | 0.7%, (N=21) | 0.4% - 1.4% |
| COPD (GOLD 1-4) | 14.6%, (N=211) | 11% - 19.1% | 14.7%, (N=224) | 12.4% - 17.3% | 10.5%, (N=190) | 8.8% - 12.4% |
| **NHANES 2011-2012** | **Normal** | **95% CI** | **Overweight** | **95% CI** | **Obesity** | **95% CI** |
| **COPD & PRISm** | |  |  |  |  |  |
| Prism | 7.1%, (N=187) | 5.7% - 8.8% | 6.8%, (N=198) | 4.9% - 9.3% | 16%, (N=348) | 13.8% - 18.6% |
| GOLD 1 | 7.7%, (N=85) | 5.8% - 10.2% | 7.4%, (N=92) | 5.7% - 9.4% | 4.5%, (N=47) | 3.2% - 6.3% |
| GOLD 2 | 6.5%, (N=83) | 4.4% - 9.4% | 6.3%, (N=87) | 4.8% - 8.3% | 6.6%, (N=89) | 4.9% - 8.8% |
| GOLD 3-4 | 1.3%, (N=20) | 0.7% - 2.4% | 1.2%, (N=14) | 0.5% - 2.9% | 1.1%, (N=18) | 0.6% - 2.1% |
| COPD (GOLD 1-4) | 15.4%, (N=188) | 12.0% - 19.6% | 14.8%, (N=193) | 12.6% - 17.5% | 12.2%, (N=154) | 10.2% - 14.6% |

**Appendix Table 5: NHANES COPD and PRISm prevalence estimates stratified by age group from 2007-2012**

| **NHANES 2007-2008** | **20-39** | **95% CI** | **40-64** | **95% CI** | **65+** | **95% CI** |
| --- | --- | --- | --- | --- | --- | --- |
| **COPD & PRISm** |  |  |  |  |  |  |
| Prism | 6.6% (N=135) | 5% - 8.8% | 11.9% (N=304) | 9.7% - 14.5% | 10.3% (N=97) | 8% - 13.2% |
| GOLD 1 | 2.6% (N=33) | 1.6% - 4.4% | 8% (N=123) | 6.5% - 9.8% | 19.5% (N=131) | 15.8% - 23.8% |
| GOLD 2 | 2.2% (N=38) | 1.5% - 3.2% | 7.9% (N=150) | 6.2% - 10.1% | 13.5% (N=122) | 10.6% - 17% |
| GOLD 3-4 | 0% (N=1) | 0% - 0.3% | 1% (N=19) | 0.5% - 2% | 3.1% (N=22) | 1.7% - 5.7% |
| COPD (GOLD 1-4) | 4.9% (N=72) | 3.2% - 7.3% | 17% (N=292) | 14% - 20.3% | 36.1% (N=265) | 32.5% - 39.9% |
| **NHANES 2009-2010** | **20-39** | **95% CI** | **40-64** | **95% CI** | **65+** | **95% CI** |
| **COPD & PRISm** |  |  |  |  |  |  |
| Prism | 8.2% (N=176) | 6.3% - 10.5% | 11% (N=319) | 8.7% - 13.7% | 8.9% (N=98) | 6.4% - 12.3% |
| GOLD 1 | 2.7% (N=43) | 1.7% - 4.2% | 9.1% (N=146) | 6.4% - 12.7% | 14.3% (N=110) | 11.2% - 18.1% |
| GOLD 2 | 1.7% (N=32) | 1.1% - 2.8% | 6.3% (N=141) | 5.1% - 7.9% | 12% (N=104) | 8.8% - 16.3% |
| GOLD 3-4 | 0.2% (N=3) | 0% - 0.7% | 0.8% (N=26) | 0.5% - 1.3% | 1.8% (N=20) | 1% - 3.2% |
| COPD (GOLD 1-4) | 4.6% (N=78) | 3.3% - 6.3% | 16.2% (N=313) | 13.2% - 19.8% | 28.1% (N=233) | 24% - 32.5% |
| **NHANES 2011-2012** | **20-39** | **95% CI** | **40-64** | **95% CI** | **65+** | **95% CI** |
| **COPD & PRISm** |  |  |  |  |  |  |
| Prism | 8.9% (N=228) | 7% - 11.2% | 11.5% (N=409) | 9.4% - 14% | 9.1% (N=96) | 6.1% - 13.5% |
| GOLD 1 | 1.5% (N=25) | 1% - 2.3% | 7.2% (N=107) | 5.6% - 9.4% | 19.1% (N=92) | 14.6% - 24.5% |
| GOLD 2 | 2% (N=40) | 1.2% - 3.4% | 8% (N=124) | 5.9% - 10.9% | 14.1% (N=95) | 10.2% - 19.2% |
| GOLD 3-4 | 0.2% (N=3) | 0.1% - 1% | 1.5% (N=29) | 0.9% - 2.5% | 2.8% (N=20) | 1.9% - 4.3% |
| COPD (GOLD 1-4) | 3.8% (N=68) | 2.7% - 5.3% | 16.8% (N=260) | 14.7% - 19.1% | 36% (N=207) | 29.7% - 42.9% |

**Appendix Table 6: NHANES COPD and PRISm prevalence estimates stratified by race/ethnicity from 2007-2012**

| **NHANES 2007-2008** | **Mexican** | **95% CI** | **Other Hispanic** | **95% CI** | **NH White** | **95% CI** | **NH Black** | **95% CI** | **Other** | **95% CI** |
| --- | --- | --- | --- | --- | --- | --- | --- | --- | --- | --- |
| **COPD & PRISm** |  |  |  |  |  |  |  |  |  |  |
| Prism | 4.2% (N=41) | 2.7% - 6.6% | 8.9% (N=45) | 6.9% - 11.3% | 6.1% (N=143) | 4.4% - 8.3% | 31.4% (N=273) | 25.8% - 37.6% | 20.4% (N=34) | 14.9% - 27.3% |
| GOLD 1 | 2.4% (N=30) | 1.7% - 3.3% | 2.2% (N=16) | 1.3% - 3.7% | 9.3% (N=213) | 8.2% - 10.5% | 1.9% (N=23) | 1.3% - 2.7% | 2.1% (N=5) | 0.8% - 5.7% |
| GOLD 2 | 1.3% (N=18) | 0.6% - 2.6% | 4% (N=21) | 2.6% - 6% | 7.1% (N=175) | 5.8% - 8.6% | 7.2% (N=78) | 5.2% - 9.8% | 3.5% (N=8) | 1.4% - 8.4% |
| GOLD 3-4 | 0.1% (N=2) | 0% - 0.6% | 0.5% (N=5) | 0.2% - 1.6% | 0.9% (N=24) | 0.5% - 1.6% | 1.1% (N=9) | 0.5% - 2.3% | 1.1% (N=2) | 0.2% - 5.7% |
| COPD (GOLD 1-4) | 3.8% (N=50) | 3% - 4.7% | 6.7% (N=42) | 4.7% - 9.5% | 17.3% (N=412) | 15.5% - 19.2% | 10.2% (N=469) | 7.9% - 13% | 6.7% (N=15) | 3.5% - 12.4% |
| **NHANES 2009-2010** | **Mexican** | **95% CI** | **Other Hispanic** | **95% CI** | **NH White** | **95% CI** | **NH Black** | **95% CI** | **Other** | **95% CI** |
| **COPD & PRISm** |  |  |  |  |  |  |  |  |  |  |
| Prism | 4.2% (N=46) | 3% - 5.8% | 7.4% (N=47) | 5.7% - 9.6% | 5.7% (N=145) | 4.3% - 7.6% | 37.4% (N=319) | 33.4% - 41.5% | 11.2% (N=27) | 7.3% - 16.9% |
| GOLD 1 | 3.6% (N=41) | 2.2% - 5.6% | 2.8% (N=20) | 1.8% - 4.2% | 9.2% (N=209) | 7.5% - 11.2% | 1.5% (N=17) | 0.9% - 2.8% | 5.3% (N=12) | 1.8% - 14.3% |
| GOLD 2 | 1.2% (N=14) | 0.6% - 2.4% | 2.5% (N=19) | 1.2% - 5.2% | 5.7% (N=158) | 4.7% - 6.9% | 6.9% (N=74) | 5.8% - 8% | 5.8% (N=12) | 2.3% - 13.6% |
| GOLD 3-4 | 0.3% (N=2) | 0.1% - 1.1% | 0.1% (N=2) | 0% - 0.6% | 0.6% (N=22) | 0.4% - 1.1% | 1.6% (N=20) | 0.9% - 2.9% | 0.7% (N=3) | 0.2% - 3% |
| COPD (GOLD 1-4) | 5% (N=57) | 3.6% - 7.1% | 5.4% (N=41) | 3.6% - 8.1% | 15.5% (N=389) | 13.2% - 18% | 10% (N=111) | 8.6% - 11.6% | 11.7% (N=3) | 5.8% - 22.3% |
| **NHANES 2011-2012** | **Mexican** | **95% CI** | **Other Hispanic** | **95% CI** | **NH White** | **95% CI** | **NH Black** | **95% CI** | **Other** | **95% CI** |
| **COPD & PRISm** |  |  |  |  |  |  |  |  |  |  |
| Prism | 4.8% (N=23) | 2.9% - 7.8% | 6.8% (N=36) | 4% - 11.4% | 5.5% (N=97) | 3.7% - 8.1% | 35.5% (N=416) | 32.1% - 39.2% | 19.6% (N=161) | 15.5% - 24.5% |
| GOLD 1 | 3.7% (N=22) | 2.6% - 5.1% | 3.5% (N=25) | 2.2% - 5.5% | 8.2% (N=127) | 6.5% - 10.2% | 1.7% (N=25) | 1.1% - 2.7% | 4.3% (N=25) | 2.1% - 8.5% |
| GOLD 2 | 1% (N=6) | 0.5% - 2% | 2.3% (N=14) | 1% - 5.1% | 7.9% (N=125) | 6.3% - 10% | 6.2% (N=89) | 5.1% - 7.4% | 3.3% (N=25) | 1.7% - 6.3% |
| GOLD 3-4 | 0% (N=0) | 0% - 0% | 0.1% (N=1) | 0% - 0.6% | 1.4% (N=22) | 1% - 2% | 1.4% (N=22) | 0.8% - 2.3% | 0.8% (N=0.8%) | 0.3% - 1.9% |
| COPD (GOLD 1-4) | 4.7% (N=28) | 3.4% - 6.3% | 5.9% (N=40) | 3.6% - 9.5% | 17.5% (N=274) | 15.2% - 20.1% | 9.3% (N=136) | 7.8% - 11% | 8.3% (N=57) | 5% - 13.6% |

**Appendix 7: Cox proportional hazard models for all-cause and cause-specific mortality by PRISm and GOLD stage with continuous age**

|  | All-Cause Mortality | | Cancer | | Cardiovascular Diseases | | Chronic Lower Respiratory Diseases | |
| --- | --- | --- | --- | --- | --- | --- | --- | --- |
|  | Hazard Ratio | 95% CI | Hazard Ratio | 95% CI | Hazard Ratio | 95% CI | Hazard Ratio | 95% CI |
| **COPD Status** |  |  |  |  |  |  |  |  |
| GOLD 0 | Ref |  | Ref |  | Ref |  | Ref |  |
| Prism | **2.1** | **(1.7 - 2.6)** | **1.8** | **(1.1 - 2.9)** | **2.2** | **(1.5 - 3.3)** | 5.0 | (1.0 - 25.5) |
| GOLD 1 | 1.0 | (0.8 - 1.3) | 1.2 | (0.7 - 1.9) | 0.8 | (0.5 - 1.2) | 1.1 | (0.3 - 4.4) |
| GOLD 2 | **1.8** | **(1.4 - 2.2)** | **1.6** | **(1.1 - 2.3)** | **2.0** | **(1.3 - 3.1)** | 2.7 | (0.7 - 9.8) |
| GOLD 3-4 | **3.5** | **(2.5 - 5.1)** | 1.0 | (0.5 - 2.1) | **4.0** | **(2.0 - 7.8)** | **44.0** | **(12.1 - 160.1)** |
| **Gender** |  |  |  |  |  |  |  |  |
| Female | Ref |  | Ref |  | Ref |  | Ref |  |
| Male | **1.8** | **(1.5 - 2.1)** | **1.8** | **(1.3 - 2.5)** | **2.3** | **(1.6 - 3.3)** | 1.8 | (0.8 - 3.7) |
| **Age** | **1.1** | **(1.1 - 1.1)** | **1.1** | **(1.1 - 1.1)** | **1.1** | **(1.1 - 1.1)** | **1.1** | **(1.1 - 1.1)** |
| **Body Mass Index** |  |  |  |  |  |  |  |  |
| Normal | Ref |  | Ref |  | Ref |  | Ref |  |
| Overweight | 0.8 | (0.7 - 1.1) | 0.8 | (0.5 - 1.3) | 1.2 | (0.8 - 2.0) | 0.5 | (0.2 - 1.6) |
| Obesity | 1.1 | (0.9 - 1.3) | 1.0 | (0.7 - 1.4) | **1.7** | **(1.1 - 2.7)** | 0.9 | (0.3 - 2.9) |
| **Smoking Status** |  |  |  |  |  |  |  |  |
| Never Smoker | Ref |  | Ref |  | Ref |  | Ref |  |
| Current Smoker | **2.3** | **(1.9 - 2.8)** | **2.6** | **(1.6 - 4.2)** | **1.8** | **(1.3 - 2.5)** | **6.6** | **(1.7 - 26.4)** |
| Former Smoker | 1.1 | (0.9 - 1.4) | 1.0 | (0.7 - 1.5) | 0.9 | (0.6 - 1.3) | 2.8 | (0.9 - 9.2) |
| **Race/Ethnicity** |  |  |  |  |  |  |  |  |
| Non-Hispanic White | Ref |  | Ref |  | Ref |  | Ref |  |
| NH Black | 1.0 | (0.8 - 1.2) | 0.9 | (0.7 - 1.3) | 1.0 | (0.7 - 1.5) | 0.3 | (0.2 - 0.7) |
| Mexican American | 0.9 | (0.7 - 1.1) | 0.7 | (0.4 - 1.2) | 1.0 | (0.6 - 1.6) | 0.4 | (0.0 - 3.4) |
| Other Hispanic | 0.9 | (0.6 - 1.2) | 0.9 | (0.6 - 1.3) | 1.1 | (0.7 - 1.9) | 0.1 | (0.0 - 1.1) |
| Other | **0.6** | **(0.4 - 0.9)** | 0.7 | (0.3 - 1.4) | 0.6 | (0.3 - 1.1) | 0.8 | (0.2 - 3.9) |
|  |  |  |  |  |  |  |  |  |
| Observations | 13,307 |  | 13,307 |  | 13,307 |  | 13,307 |  |

Values in **bold** are statistically significant.

**Appendix 8: Survival curves by mortality outcome for PRISm and any COPD**

|  |  |
| --- | --- |
| **** |  |
|  |  |

Appendix 8 presents Kaplan-Meier curves for each survival outcome by Any COPD and PRISm. Curves show the proportion of individuals still alive at a given time point. Shaded areas represent 95% confidence intervals.

**Appendix 9: Cox proportional hazard models for all-cause and cause-specific mortality by PRISm and any COPD**

|  | All-Cause Mortality | | Cancer | | Cardiovascular Diseases | | Chronic Lower Respiratory Diseases | |
| --- | --- | --- | --- | --- | --- | --- | --- | --- |
|  | Hazard Ratio | 95% CI | Hazard Ratio | 95% CI | Hazard Ratio | 95% CI | Hazard Ratio | 95% CI |
| **COPD Status** |  |  |  |  |  |  |  |  |
| GOLD 0 | Ref |  | Ref |  | Ref |  | Ref |  |
| Prism | **2.2** | **(1.8 - 2.8)** | **1.9** | **(1.2 - 3.2)** | **2.3** | **(1.5 - 3.4)** | 4.6 | (0.9 - 23.0) |
| GOLD 1-4 | **1.7** | **(1.4 - 2.1)** | **1.7** | **(1.2 - 2.4)** | **1.7** | **(1.2 - 2.4)** | **5.3** | **(1.8 - 15.5)** |
| **Gender** |  |  |  |  |  |  |  |  |
| Female | Ref |  | Ref |  | Ref |  | Ref |  |
| Male | **1.7** | **(1.5 - 1.9)** | **1.7** | **(1.3 - 2.4)** | **2.0** | **(1.4 - 3.0)** | 1.5 | (0.7 - 3.3) |
| **Age** |  |  |  |  |  |  |  |  |
| Age 20-39 | **0.3** | **(0.2 - 0.4)** | **0.1** | **(0.0 - 0.3)** | **0.2** | **(0.1 - 0.3)** | **0.1** | **(0.0 - 0.8)** |
| Age 40-64 | Ref |  | Ref |  | Ref |  | Ref |  |
| Age 65+ | **4.8** | **(4.1 - 5.6)** | **4.3** | **(3.1 - 6.0)** | **6.7** | **(4.9 - 9.1)** | **5.0** | **(2.1 - 12.1)** |
| **Body Mass Index** |  |  |  |  |  |  |  |  |
| Normal | Ref |  | Ref |  | Ref |  | Ref |  |
| Overweight | 0.9 | (0.7 - 1.1) | 0.8 | (0.5 - 1.3) | 1.3 | (0.8 - 2.1) | 0.5 | (0.2 - 1.3) |
| Obesity | 1.2 | (0.9 - 1.4) | 1.0 | (0.7 - 1.4) | **1.8** | **(1.2 - 2.8)** | 1.0 | (0.3 - 3.1) |
| **Smoking Status** |  |  |  |  |  |  |  |  |
| Never Smoker | Ref |  | Ref |  | Ref |  | Ref |  |
| Current Smoker | **2.3** | **(1.9 - 2.8)** | **2.3** | **(1.5 - 3.6)** | **1.8** | **(1.3 - 2.6)** | **9.4** | **(2.7 - 33.2)** |
| Former Smoker | 1.2 | (0.9 - 1.5) | 1.1 | (0.7 - 1.7) | 1.0 | (0.7 - 1.3) | 3.1 | (1.0 - 10.1) |
| **Race/Ethnicity** |  |  |  |  |  |  |  |  |
| Non-Hispanic White | Ref |  | Ref |  | Ref |  | Ref |  |
| NH Black | 1.0 | (0.9 - 1.3) | 0.9 | (0.6 - 1.4) | 1.1 | (0.8 - 1.6) | 0.5 | (0.2 - 1.1) |
| Mexican American | 0.8 | (0.6 - 1.0) | 0.6 | (0.4 - 1.1) | 0.9 | (0.6 - 1.4) | 0.4 | (0.0 - 2.9) |
| Other Hispanic | 0.8 | (0.6 - 1.1) | 0.8 | (0.5 - 1.2) | 1.1 | (0.6 - 1.9) | 0.1 | (0.0 - 1.0) |
| Other | **0.6** | **(0.4 - 0.9)** | 0.6 | (0.3 - 1.3) | 0.6 | (0.3 - 1.1) | 1.1 | (0.2 - 5.4) |
|  |  |  |  |  |  |  |  |  |
| Observations | 13,307 |  | 13,307 |  | 13,307 |  | 13,307 |  |

Values in **bold** are statistically significant.

**Appendix 10: Overall NHANES COPD and PRISm prevalence estimates using Hankinson’s non-Hispanic White and GLI race-neutral predictive equations**

| **Hankinson’s Predictive Equation** | **Overall 2007-2012 (N=13328)** | | **2007-2008 (N=4237)** | | **2009-2010 (N=4783)** | | **2011-2012 (N=4308)** | |
| --- | --- | --- | --- | --- | --- | --- | --- | --- |
| GOLD 0 | 76.4% (9677) | 75.2% - 77.6% | 76.2% (3072) | 74.1% - 78.1% | 77.25% (3565) | 75.2% - 79.2% | 75.8% (3040 | 73.3% - 78.1% |
| PRISm | 9.8% (1862) | 8.9% - 10.8% | 9.6% (536) | 8.1% - 11.4% | 9.6% (593) | 7.9% - 11.7% | 10.2% (733) | 8.7% - 11.9% |
| GOLD 1 | 6.9% (810) | 6.2% - 7.7% | 7.1% (287) | 6.2% - 8.3% | 7.2% (299) | 5.7% - 9.1% | 6.5% (224) | 5.4% - 7.8% |
| GOLD 2 | 6.0% (836) | 5.4% - 6.6% | 6.3% (300) | 5.2% - 7.5% | 5.3% (277) | 4.6% - 6% | 6.5% (259) | 5.3% - 7.9% |
| GOLD 3-4 | 0.9% (143) | 0.7% - 1.1% | 0.9% (42) | 0.5% - 1.4% | 0.7% (49) | 0.4% - 1.1% | 1.2% (52) | 0.8% - 1.6% |
| COPD (GOLD 1-4) | 13.8% (1789) | 12.8% - 14.9% | 14.3% (629) | 12.4% - 16.3% | 13.1% (624) | 11.2% - 15.4% | 14.1% (535) | 12.3% - 16.0% |
|  |  |  |  |  |  |  |  |  |
| **GLI Race-Neutral Predictive Equation** | **Overall 2007-2012 (N=13328)** | | **2007-2008 (N=4237)** | | **2009-2010 (N=4783)** | | **2011-2012 (N=4308)** | |
| GOLD 0 | 80.6% (10396) | 79.4% - 81.7% | 80.5% (3287) | 78.6% - 82.2% | 81.2% (3788) | 78.9% - 83.3% | 80.1% (3321) | 77.5% - 82.4% |
| PRISm | 5.6% (1143) | 4.9% - 6.4% | 5.3% (321) | 3.9% - 7.1% | 5.7% (370) | 4.6% - 6.9% | 5.9% (452) | 4.7% - 7.3% |
| GOLD 1 | 8.4% (970) | 7.6% - 9.2% | 8.8% (351) | 7.7% - 10.2% | 8.3% (350) | 6.7% - 10.3% | 8.0% (269) | 6.7% - 9.5% |
| GOLD 2 | 4.8% (707) | 4.2% - 5.4% | 4.7% (243) | 3.9% - 5.7% | 4.4% (238) | 3.7% - 5.1% | 5.3% (226) | 4.0% - 6.9% |
| GOLD 3-4 | 0.7% (112) | 0.5% - 0.9% | 0.7% (35) | 0.5% - 1.1% | 0.5% (37) | 0.3% - 0.8% | 0.8% (40) | 0.5% - 1.2% |
| COPD (GOLD 1-4) | 13.8% (1789) | 12.8% - 14.9% | 14.3% (629) | 12.4% - 16.3% | 13.1% (625) | 11.2% - 15.4% | 14.1% (535) | 12.3% - 16.0% |

| **Appendix 11: Cox proportional hazard models for all-cause mortality for NHANES 2007-2012 using the GLI race-neutral predictive equation** | | | |
| --- | --- | --- | --- |
|  | All-Cause Mortality | |  |
|  | Hazard Ratio | 95% CI |  |
| **COPD Status** |  |  |  |
| GOLD 0 | Ref |  |  |
| PRISm | **2.5** | **(2 - 3.1)** |  |
| GOLD 1 | 1.3 | (1 - 1.7) |  |
| GOLD 2 | **2.1** | **(1.7 - 2.6)** |  |
| GOLD 3-4 | **4.9** | **(3.6 - 6.6)** |  |
| **Gender** |  |  |  |
| Female | Ref |  |  |
| Male | **1.7** | **(1.5 - 2)** |  |
| **Age** |  |  |  |
| Age 20-39 | **0.3** | **(0.2 - 0.4)** |  |
| Age 40-64 | Ref |  |  |
| Age 65+ | **4.6** | **(3.9 - 5.4)** |  |
| **Body Mass Index** |  |  |  |
| Normal | Ref |  |  |
| Overweight | 0.9 | (0.7 - 1.1) |  |
| Obese | 1.2 | (1 - 1.5) |  |
| **Smoking Status** |  |  |  |
| Never Smoker | Ref |  |  |
| Current Smoker | **2.1** | **(1.7 - 2.7)** |  |
| Former Smoker | 1.1 | (0.9 - 1.4) |  |
| **Race/Ethnicity** |  |  |  |
| Non-Hispanic White | Ref |  |  |
| NH Black | 1.0 | (0.8 - 1.2) |  |
| Mexican American | 0.8 | (0.6 - 1) |  |
| Other Hispanic | 0.8 | (0.6 - 1.1) |  |
| Other | **0.6** | **(0.4 - 0.8)** |  |

**Appendix 12: All-cause mortality survival curves for COPD GOLD stage and PRISm using the GLI race-neutral predicative equation**

Appendix 12 presents the Kaplan-Meier curves for all-cause mortality by COPD GOLD stage and PRISm when using the GLI race-neutral predictive equation. Curves show the proportion of individuals still alive at a given time point. Shaded areas represent 95% confidence intervals.
